# Generalizability of the SURPASS‐2 Trial and Effect of Tirzepatide on US Diabetes and Obesity Control

**DOI:** 10.1161/JAHA.122.026297

**Published:** 2022-08-05

**Authors:** Nicholas Chiu, Rahul Aggarwal, Deepak L. Bhatt

**Affiliations:** ^1^ Department of Medicine, Beth Israel Deaconess Medical Center Harvard Medical School Boston MA; ^2^ Brigham and Women’s Hospital Heart and Vascular Center Harvard Medical School Boston MA

**Keywords:** diabetes, generalizability, obesity, tirzepatide, Diabetes, Type 2, Obesity, Cardiovascular Disease, Health Services, Quality and Outcomes

Semaglutide, a glucagon‐like peptide‐1 (GLP‐1) agonist, has been shown to reduce weight and hemoglobin A_1c_ (HbA_1c_) levels.^1^, ^2^ The SURPASS‐2 (A Study of Tirzepatide [LY3298176] Versus Semaglutide Once Weekly as Add‐on Therapy to Metformin in Participants With Type 2 Diabetes) trial recently established the superiority of tirzepatide, a dual glucose‐dependent insulinotropic polypeptide and GLP‐1 agonist, in decreasing weight and HbA_1c_, when compared with semaglutide.^1^

Given its efficacy, tirzepatide is likely to be prescribed widely. The most recent American Diabetes Association 2021 recommendations suggest the use of GLP‐1 agonists in patients with established atherosclerotic cardiovascular disease, heart failure, or chronic kidney disease independent of HbA_1c_ or metformin use.^3^ Thus, it is important to understand the SURPASS‐2 trial in the context of population generalizability and treatment effects. To better understand the US population eligible for therapy, we apply SURPASS‐2 enrollment criteria to the US population and then simulate the effects of tirzepatide on this population.

All data used are publicly available, and the data and analytical code that support the findings of this study are available from the corresponding author upon reasonable request. We applied SURPASS‐2 enrollment criteria to the National Health and Nutrition Examination Survey (2013–2018). Individuals were included if they were ≥18 years of age with type 2 diabetes, had an HbA_1c_ of 7.0% to 10.5% on metformin, and had a body mass index (BMI) ≥25 kg/m^2^. Patients were excluded if they had estimated glomerular filtration rate of <45 mL/min per 1.73 m^2^, history of diabetic retinopathy, or were taking any antidiabetes agents aside from metformin.

Baseline characteristics were determined for the US population meeting SURPASS‐2 trial criteria and compared with the trial population. We then projected the effect of 100% uptake of escalating doses of tirzepatide if BMI and HbA_1c_ reductions observed in the SURPASS‐2 trial were translated at a population level in this cohort meeting all inclusion and exclusion criteria. Based on SURPASS‐2 results, we used −2.01%, −2.24%, and −2.30% HbA_1c_ reduction values for 5‐mg, 10‐mg, and 15‐mg doses of tirzepatide, respectively; for BMI, we used −7.6‐kg, −9.3‐kg, and −11.2‐kg reductions for tirzepatide, respectively.

Given the American Diabetes Association's broad recommendations for GLP‐1 use, sensitivity analysis was additionally conducted to determine the number of eligible individuals on metformin in addition to any other antidiabetes therapy, excluding prior GLP‐1 use. A survey‐weighting design was used to determine national projections. All analyses were conducted with R version 4.0.5 (R Foundation for Statistical Computing, Vienna, Austria). National Health and Nutrition Examination Survey was approved by the National Center for Health Statistics Research Ethics Board. As part of the National Health and Nutrition Examination Survey data collection, a consent form was signed by all participants in the survey.

In our analysis, 2 991 003 (95% CI, 2 496 723–3 485 282) US adults met SURPASS‐2 criteria for initiation of tirzepatide. In sensitivity analysis permitting inclusion of individuals on prior antidiabetes agents in addition to metformin, eligible US adults increased to 4 937 063 (95% CI, 4 282 642–5 591 483).

Blood pressure and HbA_1c_ were similar between the SURPASS‐2 trial population and the eligible US population, though the eligible US population had a higher proportion of men (62.6% versus 47.0%), a higher mean weight (101.3 kg versus 93.7 kg), and a different racial and ethnic composition (Table).

**Table Table.** SURPASS‐2 Trial Generalizability and Treatment Effects on US Population

Trial population and US population meeting trial eligibility			
	SURPASS‐2 trial cohort*	US population eligible (on metformin alone)	US population eligible (Including other antidiabetic therapy)
Characteristics	n=1878	n=2 991 003 (±252 188)	n=4 937 063 (±333 894)
Age, y, mean	56.7 (±10.4)	60.4 (±0.8)	60.6 (±0.7)
Sex, %			
Male	47.0	66.4 (±4.9)	62.6% (±3.1)
Female	53.0	33.6 (±4.9)	37.4% (±3.1)
Race and ethnicity, %			
White†	82.6	63.9 (±4.3)	64.6 (±3.6)
Black	4.2	10.9 (±2.3)	10.5 (±2.0)
Hispanic‡	70.1	18.5 (±3.1)	17.6 (±2.5)
Asian	1.3	4.3 (±1.2)	4.3 (±1.0)
Other§	11.1	2.5 (±1.3)	3.0 (±1.1)
BP, mm Hg, mean			
Systolic	130.6 (±13.8)	132.3 (±1.7)	131.9 (±1.5)
Diastolic	79.2 (±9.0)	72.9 (±1.2)	71.6 (±1.0)
Weight, kg	93.7 (±21.9)	99.5 (±2.1)	100.6 (±1.7)
BMI, kg/m^2^, mean	34.2 (±6.9)	34.4 (±0.5)	34.8 (±0.5)
HbA_1c_, %, mean	8.28 (±1.0)	8.00 (±0.1)	8.07 (±0.1)
≤8.5, %	63.5%	75.1% (±3.9%)	72.4% (±3.4%)
>8.5, %	36.5%	24.9% (±3.9%)	21.0% (±3.4%)

Effect of tirzepatide on national HbA_1c_ and BMI distributions				
	Untreated US population eligible¶	Tirzepatide 5 mg	Tirzepatide 10 mg	Tirzepatide 15 mg
HbA_1c_ metrics, %				
Mean HbA_1c_	8.00 (±0.1)	5.99 (±0.1)	5.76 (±0.1)	5.70 (±0.1)
HbA_1c_ <5.7	0.0	49.6 (±4.2)	58.7 (±4.2)	58.7 (±4.2)
HbA_1c_ ≥5.7 and <6.5	0.0	25.4 (±4.3)	20.1 (±4.1)	20.1 (±4.1)
HbA_1c_ ≥6.5 and <7.0	0.0	7.6 (±2.1)	8.4 (±2.3)	8.4 (±2.3)
HbA_1c_ ≥7.0 and <8.5	73.6 (±3.9)	17.3 (±3.6)	12.7 (±3.1)	12.7 (±3.1)
HbA_1c_ ≥8.5	26.4 (±3.9)	0.0	0.0	0.0
BMI categories, kg/m^2^				
BMI, mean	34.4 (±0.5)	31.8 (±0.5)	31.2 (±0.5)	30.5 (±0.5)
BMI <18.5, %	0.0	0.0	0.0	0.0
BMI ≥18.5 and <25, %	0.0	12.4 (±2.3)	15.0 (±2.7)	19.9 (±2.9)
BMI ≥25 and <30, %	29.5 (±3.6)	39.8 (±4.0)	41.9 (±4.2)	38.7 (±4.5)
BMI ≥30, %	70.5 (±3.3)	47.8 (±4.7)	43.1 (±4.5)	41.4 (±4.2)

Demographics of SURPASS‐2 trial and US population meeting trial criteria as well as changes to HbA_1c_ and BMI distributions with 100% uptake of tirzepatide in the eligible US population. BMI indicates body mass index; HbA_1c_, hemoglobin A_1c_; and SURPASS‐2, A Study of Tirzepatide (LY3298176) Versus Semaglutide Once Weekly as Add‐on Therapy to Metformin in Participants With Type 2 Diabetes.

* Data obtained from Table 1 of article on tirzepatide.^1^

^†^ The "White" category in the SURPASS‐2 trial cohort included both Hispanic and Non‐Hispanic White individuals. In contrast, the "White" category for the US population eligible for tirzepatide as derived from the NHANES analysis includes only non‐Hispanic White individuals.

^‡^ The "Hispanic" category in the SURPASS‐2 trial cohort included both Hispanic White individuals and Non‐Hispanic White individuals. In contrast, the "White" category for the US population eligible for tirzepatide as derived from the NHANES analysis includes only non‐White Hispanic individuals.

^§^ The "Other" category in the SURPASS‐2 trial cohort refers to American Indian or Alaska Native individuals. In contrast, the "Other" category for the US population eligible for tirzepatide as derived from the NHANES analysis refers to non‐Hispanic persons reporting races other than Black, Asian, or White. This includes Multi‐Racial as designated in NHANES.

^¶^ The “Untreated US Population Eligible” refers to the 2 991 003 projected individuals eligible for tirzepatide by SURPASS‐2 Trial criteria on metformin alone; the HbA_1c_ and BMI metrics for this column are assuming no treatment with tirzepatide.

In the US eligible population, complete uptake of 15 mg tirzepatide was simulated to reduce mean A_1c_ from to 8.00% to 5.70% and decrease the proportion of obese individuals from 70.5% to 41.4%. Changes in national A_1c_ and BMI distributions by tirzepatide dose are displayed in the Table.

With ≈3.0 million adults eligible for tirzepatide, and up to ≈4.9 million on sensitivity analysis, our results demonstrate that SURPASS‐2 criteria are widely generalizable. Strikingly, complete uptake of tirzepatide in the eligible US population could result in significant changes to the national distribution of HbA_1c_ and BMI.

Despite the promise of therapy, we found that over half of eligible adults were not on any antidiabetes medications aside from metformin despite a mean HbA_1c_ of 8.0%, and a quarter of individuals with HbA_1c_ >8.5%. Our results underscore continued challenges in diabetes care, where multiple effective agents exist—though the US population continues to struggle with uptake. The introduction of novel agents such as tirzepatide have the potential to greatly improve diabetes control; however, approaches to ensure population uptake are urgently needed.

It is important to note clinically that in SURPASS‐2, adverse events leading to discontinuation of tirzepatide occurred in 6% of patients on a 5‐mg dose and up to 8.5% for those on a 10‐mg or 15‐mg dose. This is in contrast to 4.1% of patients in the comparison semaglutide 1‐mg group. Allowing a conservative estimate, if ≈10% of patients discontinued tirzepatide on the basis of side effects, only ≈2.7 million of the projected ≈3.0 million adults could tolerate maximum doses. Thus, side effects on tirzepatide must be monitored clinically, and clinicians should individualize therapy choices based on patients' responses.

There are several limitations to our study. First, survey data are subject to response bias. Second, we do factor cost into consideration, and our simulation on treatment effects does not take into account treatment heterogeneity of the US population.

In conclusion, tirzepatide is broadly generalizable, and increased prescription could have large effects on the control of diabetes and obesity.

## Sources of Funding

None.

## Disclosures

Dr Bhatt discloses the following relationships: Advisory Board: AngioWave, Bayer, Boehringer Ingelheim, Cardax, CellProthera, Cereno Scientific, Elsevier Practice Update Cardiology, High Enroll, Janssen, Level Ex, Medscape Cardiology, Merck, MyoKardia, NirvaMed, Novo Nordisk, PhaseBio, PLx Pharma, Regado Biosciences, and Stasys; Board of Directors: AngioWave (stock options), Boston VA Research Institute, Bristol Myers Squibb (stock), DRS.LINQ (stock options), High Enroll (stock), Society of Cardiovascular Patient Care, and TobeSoft; Chair: Inaugural Chair, American Heart Association Quality Oversight Committee; Data Monitoring Committees: Acesion Pharma, Assistance Publique‐Hôpitaux de Paris, Baim Institute for Clinical Research (formerly Harvard Clinical Research Institute, for the PORTICO trial, funded by St. Jude Medical, now Abbott), Boston Scientific (Chair, PEITHO trial), Cleveland Clinic (including for the ExCEED trial, funded by Edwards), Contego Medical (Chair, PERFORMANCE 2), Duke Clinical Research Institute, Mayo Clinic, Mount Sinai School of Medicine (for the ENVISAGE trial, funded by Daiichi Sankyo; for the ABILITY‐DM trial, funded by Concept Medical), Novartis, and Population Health Research Institute; Rutgers University (for the NIH‐funded MINT Trial); Honoraria: American College of Cardiology (Senior Associate Editor, Clinical Trials and News, ACC.org; Chair, ACC Accreditation Oversight Committee), Arnold and Porter law firm (work related to Sanofi/Bristol‐Myers Squibb clopidogrel litigation), and Baim Institute for Clinical Research (formerly Harvard Clinical Research Institute); RE‐DUAL PCI clinical trial steering committee funded by Boehringer Ingelheim; AEGIS‐II executive committee funded by CSL Behring), Belvoir Publications (Editor in Chief, Harvard Heart Letter), Canadian Medical and Surgical Knowledge Translation Research Group (clinical trial steering committees), Cowen and Company, Duke Clinical Research Institute (clinical trial steering committees, including for the PRONOUNCE trial, funded by Ferring Pharmaceuticals), HMP Global (Editor in Chief, *Journal of Invasive Cardiology*), *Journal of the American College of Cardiology* (Guest Editor; Associate Editor), K2P (Co‐Chair, interdisciplinary curriculum), Level Ex, Medtelligence/ReachMD (CME steering committees), MJH Life Sciences, Oakstone CME (Course Director, Comprehensive Review of Interventional Cardiology), Piper Sandler, Population Health Research Institute (for the COMPASS operations committee, publications committee, steering committee, and USA national co‐leader, funded by Bayer), Slack Publications (Chief Medical Editor, *Cardiology Today*'*s Intervention*), Society of Cardiovascular Patient Care (Secretary/Treasurer), WebMD (CME steering committees), and Wiley (steering committee); Other: *Clinical Cardiology* (Deputy Editor), NCDR‐ACTION Registry Steering Committee (Chair), and VA CART Research and Publications Committee (Chair); Research Funding: Abbott, Acesion Pharma, Afimmune, Aker Biomarine, Amarin, Amgen, AstraZeneca, Bayer, Beren, Boehringer Ingelheim, Boston Scientific, Bristol‐Myers Squibb, Cardax, CellProthera, Cereno Scientific, Chiesi, CSL Behring, Eisai, Ethicon, Faraday Pharmaceuticals, Ferring Pharmaceuticals, Forest Laboratories, Fractyl, Garmin, HLS Therapeutics, Idorsia, Ironwood, Ischemix, Janssen, Javelin, Lexicon, Lilly, Medtronic, Merck, Moderna, MyoKardia, NirvaMed, Novartis, Novo Nordisk, Owkin, Pfizer, PhaseBio, PLx Pharma, Recardio, Regeneron, Reid Hoffman Foundation, Roche, Sanofi, Stasys, Synaptic, The Medicines Company, and 89Bio; Royalties: Elsevier (Editor, *Braunwald*'*s Heart Disease*); Site Co‐Investigator: Abbott, Biotronik, Boston Scientific, CSI, Endotronix, St. Jude Medical (now Abbott), Philips, Svelte, and Vascular Solutions; Trustee: American College of Cardiology; Unfunded Research: FlowCo, Takeda. The remaining authors have no disclosures to report.

## References

[1] Frías JP , Davies MJ , Rosenstock J , Pérez Manghi FC , Fernández Landó L , Bergman BK , Liu B , Cui X , Brown K . Tirzepatide versus semaglutide once weekly in patients with type 2 diabetes. N Engl J Med. 2021;385:503–515. doi: 10.1056/NEJMoa2107519 34170647

[2] Davies M , Færch L , Jeppesen OK , Pakseresht A , Pedersen SD , Perreault L , Rosenstock J , Shimomura I , Viljoen A , Wadden TA , et al; STEP 2 Study Group . Semaglutide 2·4 mg once a week in adults with overweight or obesity, and type 2 diabetes (STEP 2): a randomised, double‐blind, double‐dummy, placebo‐controlled, phase 3 trial. Lancet. 2021;397:971–984. doi: 10.1016/S0140-6736(21)00213-0. 33667417

[3] Association AD . 9. Pharmacologic Approaches to Glycemic Treatment: Standards of Medical Care in Diabetes—2021. Diabetes Care. 2021;44:S111–S124. doi: 10.2337/dc21-S009 33298420

